# Localization of obstruction sites in obstructive azoospermia: role of combined transscrotal–transrectal ultrasonography

**DOI:** 10.1186/s13244-025-02143-x

**Published:** 2025-12-02

**Authors:** Xin Li, Chen-Cheng Yao, Chen-Wang Zhang, Xiao-Bo Wang, Li-Ren Jiang, Zheng Li, Peng Li, Rong Wu

**Affiliations:** 1 Department of Ultrasound, Shanghai General Hospital, Shanghai Jiao Tong University School of Medicine, Shanghai, China; 2 Department of Andrology, The Center for Men’s Health, Urologic Medical Center, Shanghai Key Laboratory of Reproductive Medicine, Shanghai General Hospital, Shanghai Jiao Tong University School of Medicine, Shanghai, China; 3 State Key Lab of Reproductive Medicine and Offspring Health, Nanjing Medical University, Nanjing, China; 4 Department of Pathology, Shanghai General Hospital, Shanghai Jiao Tong University School of Medicine, Shanghai, China; 5 Department of Urology, Ningde Municipal Hospital of Ningde Normal University, Ningde, China

**Keywords:** Obstructive azoospermia, Transscrotal–transrectal ultrasonography, Obstructive site

## Abstract

**Objective:**

To evaluate the diagnostic performance of combined transscrotal–transrectal ultrasonography in predicting sites of obstructive azoospermia.

**Materials and methods:**

From June 2019 to March 2023, 166 obstructive azoospermia patients who underwent surgical exploration were enrolled in the retrospective study. The data of combined transscrotal–transrectal ultrasonography in 166 patients were collected and analyzed. The receiver operating characteristic (ROC) curve analysis was employed to evaluate the diagnostic performance of these ultrasonographic measurements for localizing different obstructive sites.

**Results:**

There were 9 sides of intratesticular obstruction, 239 sides of epididymal obstruction, 68 sides of vas deferens obstruction, and 16 sides of ejaculatory duct obstruction. The sensitivity, specificity, and the area under the curve (AUC) for combined transscrotal–transrectal ultrasonography were 44.4%, 98.5% and 0.714 for diagnosing intratesticular obstruction; 97.9%, 84.9% and 0.919 for diagnosing epididymal obstruction; 82.4%, 99.2% and 0.913 for diagnosing vas deferens obstruction; and 87.5%, 99.1% and 0.93 for diagnosing ejaculatory duct obstruction. The sensitivity, specificity, and AUC were 88.9%, 83.9% and 0.842 in diagnosing intratesticular obstruction for a rete testis thickness cut-off of 3.0 mm; 81.0%, 100% and 0.949 in diagnosing vas deferens obstruction for a 0.8 mm cutoff for the internal diameter of the scrotal section of the vas deferens; and 62.5%, 92.6% and 0.769 in diagnosing ejaculatory duct obstruction for a seminal vesicle diameter cut-off of 12.5 mm.

**Conclusion:**

Combined transscrotal–transrectal ultrasonography, evaluating specific structures of rete testis thickness, seminal vesicle diameter, and the internal diameter of the scrotal vas deferens, could accurately localize obstruction sites in obstructive azoospermia patients.

**Critical relevance statement:**

Combined transscrotal–transrectal ultrasonography demonstrated high diagnostic performance in predicting the sites of epididymal, vas deferens, and ejaculatory duct obstruction in patients with obstructive azoospermia.

**Key Points:**

- The diagnostic performance of combined transscrotal–transrectal ultrasonography in obstructive azoospermia was evaluated.
- Ultrasound measurements of specific structures significantly improve the prediction of obstruction sites.
- Combined transscrotal–transrectal ultrasonography accurately localizes obstruction sites in obstructive azoospermia patients.

**Graphical Abstract:**

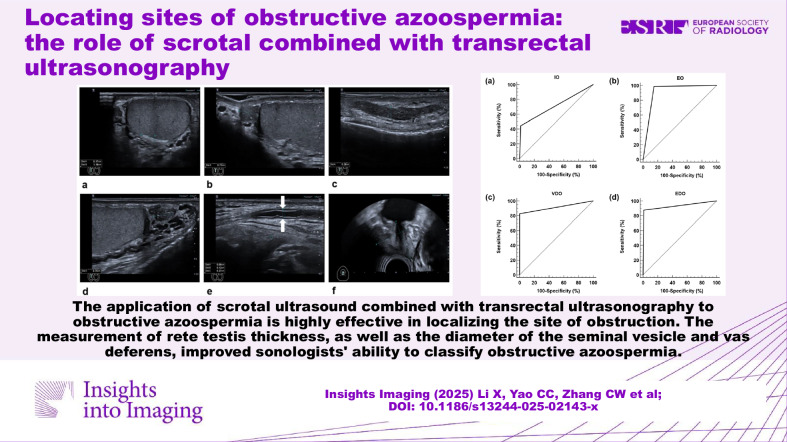

## Introduction

Obstructive azoospermia (OA) accounts for approximately 40% of all azoospermia cases [1–3]. The obstructive site(s) of OA can be found in any part of the male reproductive tract, such as the intratesticular, epididymal, vas deferens, and ejaculatory duct [4]. The surgical approach varies depending on the location of the obstruction in OA patients. For example, epididymal obstruction (EO) requires vasoepididymostomy (VE), while intratesticular obstruction (IO) requires testicular sperm extraction and intracytoplasmic sperm injection (ICSI) [5, 6]. Therefore, preoperative determination of the obstruction’s location is important for planning surgical strategies for OA patients.

Vasography was once considered a reference standard for locating sites of OA and especially as the gold standard for diagnosing proximal and distal ejaculatory duct obstruction (EDO) [7, 8]. However, as an invasive examination, it not only has a certain puncture failure rate but also causes damage to the puncture site or secondary infection, which may lead to secondary obstruction of the seminal tract. Therefore, developing an effective, non-invasive diagnostic method for locating sites of OA is of great significance.

Ultrasonography is an important diagnostic tool for evaluating male infertility [9]. Transscrotal ultrasonography, a specific type of ultrasound, helps assess testicular volume (TV) and indicate obstructive factors in azoospermia by displaying net-like ectasia of the rete testis and epididymis, as well as the dilation of the scrotal segment of the vas deferens [2, 10–13]. Transrectal ultrasonography could also indicate obstructive factors in azoospermia by the non-display of the terminal segment of the vas deferens and the presence of cysts in the ejaculatory duct area[2, 8, 14–16]. Previous studies have focused on the ultrasound characteristics of a single obstruction site in OA patients. However, a comprehensive analysis of the entire seminal tract is crucial for accurately determining the obstruction site and aiding in clinical decision-making. Therefore, the aim of this study was to evaluate the diagnostic performance of combined transscrotal–transrectal ultrasonography as a comprehensive scanning protocol for localizing obstruction sites in OA patients.

## Materials and methods

### Participants

This retrospective study was approved by the Ethics Committee of Shanghai General Hospital, Shanghai Jiao Tong University School of Medicine (2020SQ333), and was conducted in accordance with the principles of the Helsinki Declaration. Written informed consent was waived due to the retrospective nature of the study.

**Fig. 1:**
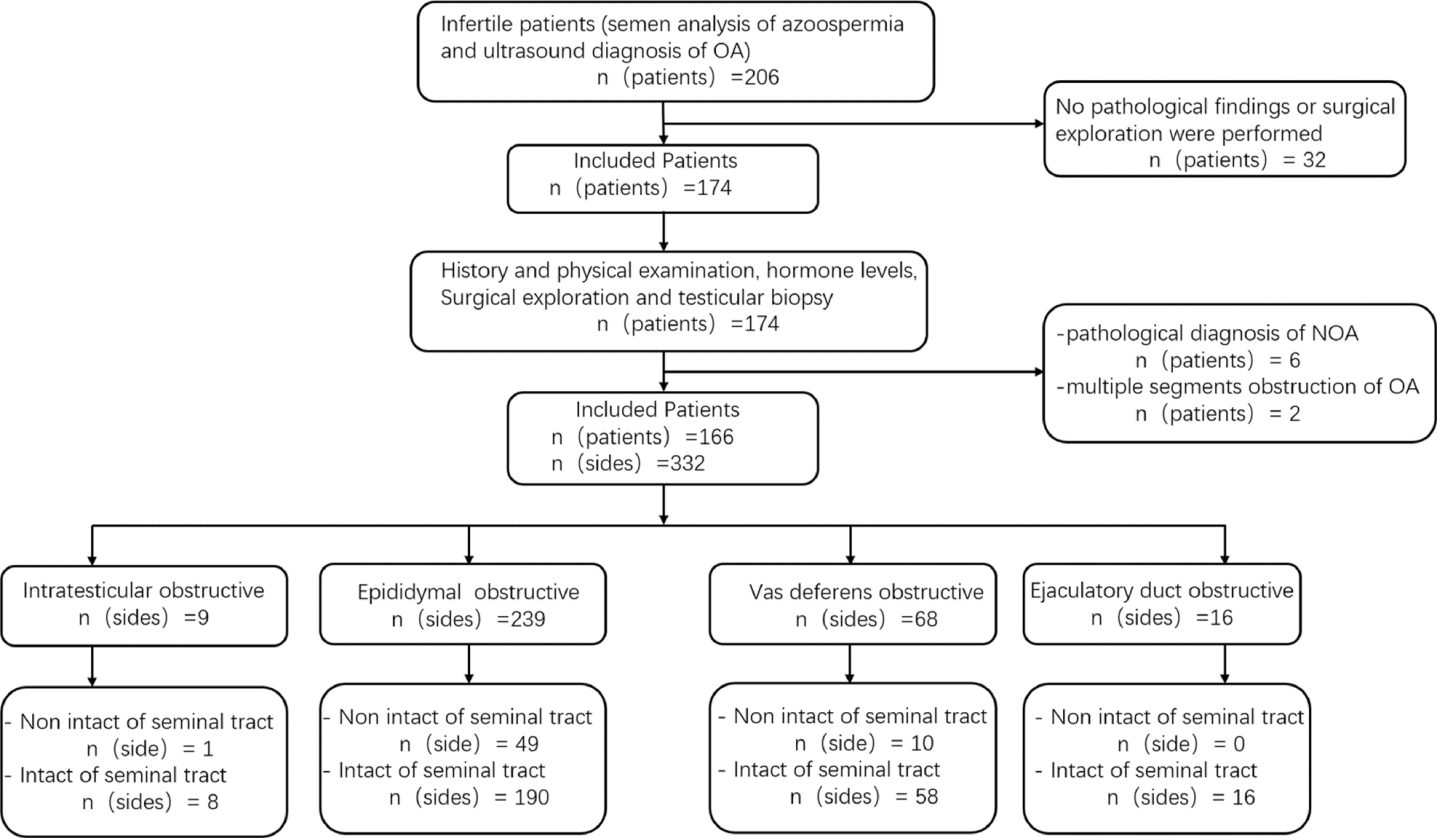
Patient enrollment and classification flowchart

From June 2019 to March 2023, 206 infertile patients with potential OA were initially enrolled in the study. Of these, 32 patients were excluded because they lacked final pathological results. From the remaining 174 patients, an additional 6 patients were excluded after a pathological diagnosis of non-obstructive azoospermia (NOA), and 2 patients with multiple obstructive sites were also excluded. Finally, 166 OA patients (332 sides) with a single obstructive site were included. The study’s flowchart detailing these inclusion and exclusion criteria is presented in Fig. 1.

### Ultrasonographic technique

Transscrotal and transrectal ultrasonography were performed using Canon Medical Systems scanners (Aplio 500/900 with 5–14/18 MHz linear-array transducers for scrotal imaging; Aplio i500 with 3–11 MHz intracavity transducer for rectal evaluation).

Patients were placed in a supine position with the penis secured against the abdomen and the testes fully exposed. The bilateral TV was calculated using Lambert’s formula: length × height × width × 0.71 [17]. To measure rete testis thickness (RTT), the anterior-posterior diameter of the thickest part of the rete testis was measured from a longitudinal ultrasound section, and the average of five measurements was recorded. All RTT measurements were completed at the time of image acquisition [18]. A linear-array transducer was used to fully visualize the epididymis with imaging planes perpendicular to its surface, as well as the scrotal segments of the vas deferens. Thickness measurements were taken at the enlarged parts of the epididymal caput, mid-epididymal corpus, cauda, and the luminal diameter between the vas deferens walls. Subsequently, axial and sagittal scans were performed to evaluate the terminal segments of the pelvic vas deferens, seminal vesicles, and the ejaculatory duct area. The seminal vesicle diameter (SVD) was measured as the anteroposterior dimension on axial planes.

All examinations were performed by a single skilled radiologist (X.L.) with 15 years of experience in conventional ultrasound. The images were recorded on a hard disk incorporated in the scanner.

### Imaging analysis for localizing obstructive sites in OA

Ultrasonography determines the obstructive site of OA by identifying the farthest point of semen blockage along the outflow path of the seminal tract. The diagnostic criteria for each site are as follows: (1) IO: (a) absent epididymal caput, with normal/absent ipsilateral reproductive ducts, or (b) rete testis net-like ectasia, no obstruction in other ipsilateral reproductive ducts. (2) EO: epididymal net-like ectasia or absent corpus/cauda, and normal ipsilateral testis; distal reproductive ducts (vas deferens, ejaculatory duct, seminal vesicles) normal/absent. The site of obstruction in the epididymis was further determined. (a) Epididymal caput obstruction (EO-Cap): epididymal caput net-like ectasia, with normal/absent ipsilateral epididymal corpus and cauda. (b) Epididymal corpus obstruction (EO-Cor): epididymal corpus net-like ectasia, with normal/absent ipsilateral epididymal cauda. (c) Epididymal cauda obstruction (EO-Cau): epididymal cauda net-like ectasia, with normal/absent ipsilateral vas deferens. (3) Vas deferens obstruction (VDO): scrotal segment of the vas deferens dilation, with normal or net-like ectasia ipsilateral testis and epididymis, and normal or absent ipsilateral distal reproductive duct of vas deferens. (4) EDO: ejaculatory duct cysts, with normal ipsilateral testes, epididymis, vas deferens, and seminal vesicles, or seminal vesicles dilation. Ultrasound diagnosis located the obstructive site of OA in the ejaculatory duct area. Since transrectal ultrasound cannot distinguish between the left and right cysts of the ejaculatory ducts [2], when the ultrasound diagnosis identified OA obstruction in the ejaculatory duct area, it was considered a bilateral EDO (Supplement Figs. 1–6).

### Reference standard

Azoospermia was confirmed following the guidelines of the fifth edition of the World Health Organization laboratory manual for the examination and processing of human semen [19]. The diagnosis of OA was made through bilateral testicular biopsy. The specific obstructive site was identified via surgical exploration. Histology assessments were performed by two pathologists, X.-B.W. and L.-R.J., with 16 years and 7 years of work experience, respectively. In case of disagreement, it was necessary to reach a consensus through further discussion between the two doctors. The histology criteria used in this study followed previously reported criteria in assessing azoospermia [18, 20].

### Statistical analysis

The age and sex hormone levels, including the follicle-stimulating hormone (FSH), luteinizing hormone (LH), testosterone (T), TV, and RTT of OA patients with four different obstructive sites were expressed as mean ± standard deviation (SD). Analysis of variance (ANOVA) was used to compare groups after ensuring homogeneity of variances for the data. The Kruskal–Wallis test compared the age, sex hormone levels, TV, and RTT among the groups. The receiver operating characteristic (ROC) curve analysis was used to evaluate the diagnostic performance of transscrotal–transrectal ultrasonography in the diagnosis of different obstructive sites in OA patients. Moreover, the area under the curve (AUC) and its 95% confidence interval (CI) were calculated. The epididymal caput thickness (ET-Cap), epididymal corpus thickness (ET-Cor), and epididymal cauda thickness (ET-Cau) were expressed as mean ± SD when the seminal tract was intact and compared among the groups using ANOVA after homogeneity of variances for the data. When the Kruskal–Wallis test results showed significant statistical significance (*p* < 0.05), the Wilcoxon signed-rank test was used to compare every two groups. Inner diameter of the scrotal segment of the vas deferens (IDSSVD) and SVD were represented by the median (interquartile range [IQR]). The ROC curve was used to evaluate the diagnostic performance of RTT for IO, ET-Cor for EO-Cor, ET-Cau for EO-Cau, and IDSSVD for VDO. The diagnostic performance of SVD for EDO was also calculated, along with AUC and its 95% CI. *p* < 0.05 was considered statistically significant. The statistical software SPSS 26.0 (IBM Corp.) was used for statistical analysis. The diagnostic performance of the ROC curves was compared using the MedCalc 19.2.0 software program (MedCalc Software Ltd.).

## Results

### Basic characteristics of enrolled patients

The mean age of the IO, EO, VDO, and EDO groups was 32 ± 10 years (range: 22–50 years), 32 ± 6 years (range: 20–57 years), 31 ± 6 years (range: 21–57 years), and 35 ± 4 years (range: 30–41 years), respectively. There was no statistically significant difference in age distribution among the four groups (*p* > 0.05). The same applied to sex hormone levels (including FSH, LH, and T) and TV among the four groups of patients (*p* > 0.05). Meanwhile, the RTT of the IO patients was significantly higher than that of EO (*p* < 0.001), VDO (*p* < 0.001), and EDO (*p* = 0.002) patients. However, no statistically significant difference in RTT was noted between EO, VDO, and EDO (*p* > 0.05, Table 1).

**Table 1:** Comparison of patient characteristics, hormones, TV, RTT, and diagnostic performance in identifying different obstructive sites in patients by transscrotal–transrectal ultrasonography

Variable	IO *n* (sides) = 9	EO *n* (sides) = 239	VDO *n* (sides) = 68	EDO *n* (sides) = 16
Age (y) mean ± SD	32 ± 10	32 ± 6	31 ± 6	35 ± 4
FSH (IU/L) mean ± SD	4.5 ± 1.3	4.8 ± 3.4	4.8 ± 2.4	3.5 ± 2.4
LH (IU/L) mean ± SD	4.3 ± 1.2	5.3 ± 3.3	5.2 ± 2.4	4.1 ± 2.1
T (ug/ L) mean ± SD	3.8 ± 1.0	3.9 ± 1.5	4.1 ± 1.7	4.0 ± 1.8
TV (cm^3^) mean ± SD	15.7 ± 3.1	14.9 ± 3.7	14.7 ± 3.7	16.0 ± 3.2
RTT (mm) mean ± SD	3.6 ± 1.1^a,b,c^	2.4 ± 0.9^a^	2.4 ± 0.7^b^	2.5 ± 0.6^c^
SEN (%)	44.4	97.9	82.4	87.5
SPE (%)	98.5	84.9	99.2	99.1
AUC	0.714 (0.500–0.929)	0.919 (0.876–0.963)	0.913 (0.860–0.966)	0.933 (0.837–1.000)

*IO* intratesticular obstruction, *EO* epididymal obstruction, *VDO* vas deferens obstruction, *EDO* ejaculatory duct obstruction, *SD* standard deviation, *FSH* follicle-stimulating hormone, *LH* luteinizing hormone, *T* testosterone, *TV* testicular volume, *RTT* rete testis thickness, *SEN* sensitivity, *SPE* specificity, *AUC* area under receiver operating characteristic curve

^a^Indicates a significant difference between IO and EO (*p* < 0.001)

^b^Indicates a significant difference between IO and VDO (*p* < 0.001)

^c^Indicates a significant difference between IO and EDO (*p* = 0.002)

### Diagnostic performance of transscrotal–transrectal ultrasonography for OA

**Fig. 2:**
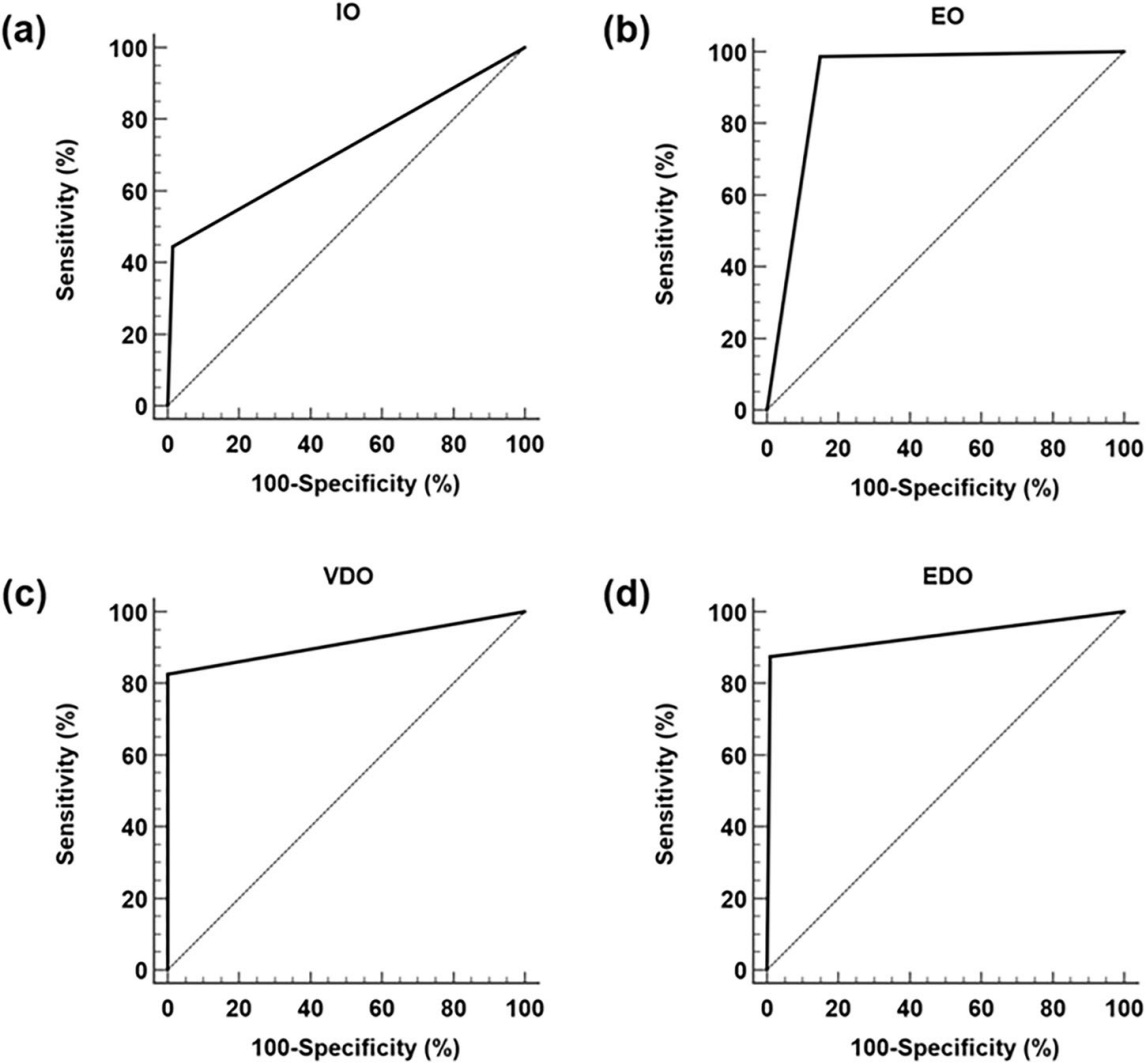
Evaluation of ROC curves at four different obstructive sites using transscrotal–transrectal ultrasonography. **a** ROC curve for evaluating the performance of IO diagnosis using transscrotal–transrectal ultrasonography. **b** ROC curve for evaluating the performance of EO diagnosis using transscrotal–transrectal ultrasonography. **c** ROC curve for evaluating the performance of VDO diagnosis using transscrotal–transrectal ultrasonography. **d** ROC curve for evaluating the performance of EDO diagnosis using transscrotal–transrectal ultrasonography

The sensitivity, specificity, and AUC of transscrotal–transrectal ultrasonography were as follows: 44.4%, 98.5%, and 0.714 (0.500–0.929), respectively, for diagnosing IO; 97.9%, 84.9%, and 0.919 (0.876–0.963), respectively, for EO; 82.4%, 99.2%, and 0.913 (0.860–0.966), respectively, for VDO; and 87.5%, 99.1%, and 0.933 (0.837–1.000), respectively, for EDO (Fig. 2).

### Comparison of epididymis thickness, IDSSVD, and SVD among different groups

When the seminal tract was intact, there were 8 sides of IO, 190 sides of EO, 58 sides of VDO, and 16 sides of EDO. The 190 sides of EO included 35 sides with EO-Cap, 46 sides with EO-Cor, and 109 sides with EO-Cau. The results showed that (1) ET-Cap of EO-Cap and EO-Cor were greater than that of EO-Cau (EO-Cap: *p* = 0.020, EO-Cor: *p* = 0.001). (2) The ET-Cor of IO was smaller than that of EO-Cor, EO-Cau, and VDO (EO-Cor: *p* = 0.001, EO-Cau: *p* < 0.001, VDO: *p* = 0.001); the ET-Cor of EO-Cap was smaller than that of EO-Cor, EO-Cau, and VDO (EO-Cor: *p* = 0.001, EO-Cau: *p* < 0.001, VDO: *p* = 0.001); the ET-Cor of EO-Cor, EO-Cau, and VDO were all greater than that of EDO (All *p* < 0.001). (3) The ET-Cau of EO-Cap, EO-Cor, EO-Cau, and EDO were all smaller than that of VDO (EO-Cap: *p* = 0.023, EO-Cor: *p* < 0.001, EO-Cau: *p* < 0.001, EDO: *p* = 0.003). (4) The IDSSVD of IO, EO-Cap, EO-Cor, EO-Cau, and EDO were all smaller than that of VDO (all *p* < 0.001). (5) The SVD of IO, EO-Cap, EO-Cor, EO-Cau, and VDO was smaller than that of EDO (IO: *p* = 0.028, EO-Cap: *p* < 0.001, EO-Cor: *p* = 0.001, EO-Cau: *p* < 0.001, VDO: *p* = 0.019) (Table 2).

**Table 2:** Comparison of epididymis thickness, seminal vesicle diameter, and internal diameter of the scrotal segment of the vas deferens among different groups when the seminal tract was intact

Variable	IOn (sides) = 8	EO *n* (sides) = 190			VDO *n* (sides) = 58	EDO *n* (sides) = 16
		EO-Cap n (sides) = 35	EO-Cor *n* (sides) = 46	EO-Cau *n* (sides) = 109		
ET-Cap (mm) mean ± SD	9.0 ± 3.0	9.2 ± 2.6^a^	9.6 ± 2.1^b^	8.3 ± 2.1^a,b^	8.8 ± 1.8	8.5 ± 1.8
ET-Cor (mm) mean ± SD	3.2 ± 0.9^c,d,e^	3.8 ± 1.1^a,f,g^	4.7 ± 1.3^c,f,h^	4.9 ± 1.2^a,d,i^	4.6 ± 1.0^e,g,j^	2.9 ± 1.1 ^h,i,j^
ET-Cau (mm) mean ± SD	7.8 ± 3.5	7.7 ± 1.8^g^	7.1 ± 2.0^k^	7.3 ± 2.1^l^	8.7 ± 2.1^j,g,k,l^	7.0 ± 1.4^j^
IDSSVD (mm) median (IQR)	0.5 (0.4, 0.6)^e^	0.5 (0.5, 0.6)^g^	0.5 (0.4, 0.5)^k^	0.5 (0.4, 0.6)^l^	1.3 (0.9, 1.7)^e,j,g,k,l^	0.5 (0.4, 0.5)^j^
SVD (mm) median (IQR)	9.0 (8.3, 10.5)^m^	9.0 (7.6, 10.0)^n^	9.0 (8.6, 10.0)^h^	9.0 (8.0, 10.0)^i^	10.0 (8.0, 12.0)^j^	14.5 (10.0, 18.3)^h,i,j,m,n^

*IO* intratesticular obstruction, *EO* epididymal obstruction, *EO-Cap* epididymal caput obstruction, *EO-Cor* epididymal corpus obstruction, *EO-Cau* epididymal cauda obstruction, *VDO* vas deferens obstruction, *EDO* ejaculatory duct obstruction, *ET-Cap* epididymal caput thickness, *ET-Cor* epididymal corpus thickness, *ET-Cau* epididymal cauda thickness, *SD* standard deviation, *IDSSVD* internal diameter of scrotal section of Vas deferens, *IQR* interquartile range, *SVD* seminal vesicle diameter

^a^Indicates a significant difference between EO-Cap and EO-Cau (ET-Cap: *p* = 0.020, ET-Cor: *p* < 0.001)

bIndicates a significant difference between EO-Cor and EO-Cau (ET-Cap: *p* = 0.001)

^c^Indicates a significant difference between IO and EO-Cor (ET-Cor: *p* = 0.001)

^d^Indicates a significant difference between IO and EO-Cau (ET-Cor: *p* < 0.001)

^e^Indicates a significant difference between IO and VDO (ET-Cor: *p* = 0.001, IDSSVD: *p* < 0.001)

^f^Indicates a significant difference between EO-Cap and EO-Cor (ET-Cor: *p* = 0.001)

^g^Indicates a significant difference between EO-Cap and VDO (ET-Cor: *p* = 0.001, ET-Cau: *p* = 0.023, IDSSVD: *p* < 0.001)

^h^Indicates a significant difference between EO-Cor and EDO (ET-Cor: *p* < 0.001, SVD: *p* = 0.001)

^i^Indicates a significant difference between EO-Cau and EDO (ET-Cor: *p* < 0.001, SVD: *p* < 0.001)

^j^Indicates a significant difference between VDO and EDO (ET-Cor: *p* < 0.001, ET-Cau: *p* = 0.003, IDSSVD: *p* < 0.001, SVD: *p* = 0.019)

^k^Indicates a significant difference between EO-Cor and VDO (ET-Cau: *p* < 0.001, IDSSVD: *p* < 0.001)

^l^Indicates a significant difference between EO-Cau and VDO (ET-Cau: *p* < 0.001, IDSSVD: *p* < 0.001)

^m^Indicates a significant difference between IO and EDO (SVD: *p* = 0.028)

^n^Indicates a significant difference between EO-Cap and EDO (SVD: *p* < 0.001)

### Diagnostic performance of RTT, ET-Cor, ET-Cau, IDSSVD, and SVD

**Fig. 3:**
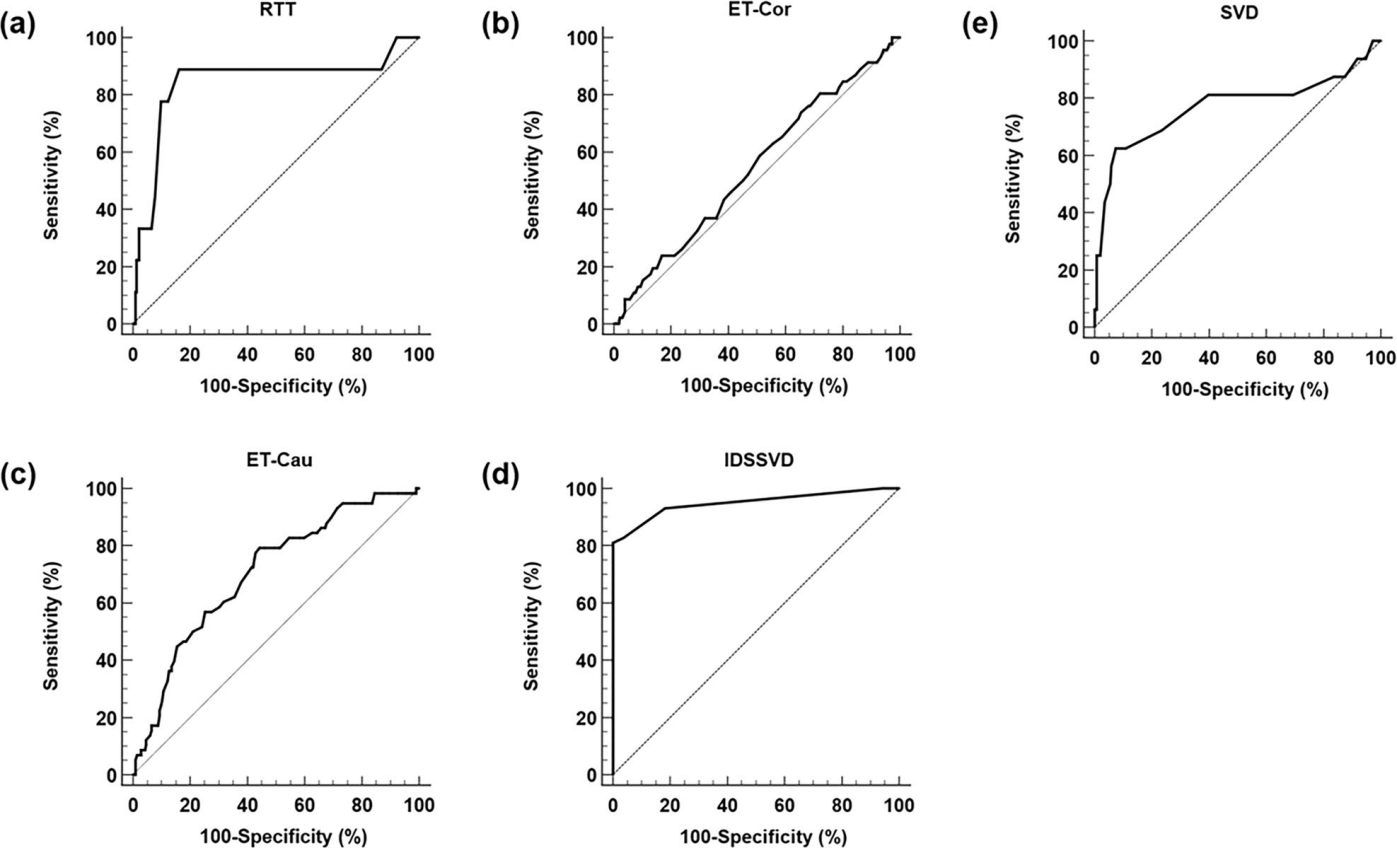
ROC curve. **a** ROC curve for evaluating the diagnostic performance of IO based on RTT. **b** ROC curve for evaluating the diagnostic performance of EO-Cor based on ET-Cor. **c** ROC curve for evaluating the diagnostic performance of EO-Cau based on ET-Cau. **d** ROC curve for evaluating the diagnostic performance of VDO based on IDSSVD. **e** ROC curve for evaluating the diagnostic performance of EDO using SVD. IO, intratesticular obstruction; RTT, rete testis thickness; EO-Cor, epididymal corpus obstruction; ET-Cor, epididymal corpus thickness; EO-Cau, epididymal cauda obstruction; ET-Cau, epididymal cauda thickness; VDO, vas deferens obstruction; IDSSVD, internal diameter of scrotal section of vas deferens; EDO, ejaculatory duct obstruction; SVD, seminal vesicle diameter

The sensitivity, specificity, and AUC were 88.9%, 83.9%, and 0.842 (95% CI: 0.668–1.000), respectively, for an RTT cut-off of 3.0 mm for diagnosing IO; 73.9%, 34.5%, and 0.543 (95% CI: 0.452–0.634), respectively, for an ET-Cor cut-off of 4.1 mm for diagnosing EO-Cor; 79.3%, 65.6%, and 0.704 (95% CI: 0.630–0.777), respectively, for an ET-Cau cut-off of 7.5 mm for diagnosing EO-Cau; 81.0%, 100%, and 0.949 (95% CI: 0.909–0.990), respectively, for an IDSSVD cut-off of 0.8 mm for diagnosing VDO; and 62.5%, 92.6%, and 0.769 (95% CI: 0.608–0.930), respectively, for an SVD cut-off of 12.5 mm for diagnosing EDO (Fig. 3).

## Discussion

In our study, we excluded patients with multiple-segment obstruction due to a small sample size. This type of obstruction falls under complex seminal tract obstruction, which cannot be definitively diagnosed pre-surgically and requires intraoperative exploration to pinpoint the site of obstruction. Therefore, all OA patients included in this study had single-site obstruction.

This study found that the specificity of transscrotal–transrectal ultrasonography in evaluating IO was high, reaching 98.5%, but its sensitivity was a lowly 44.4%. Reviewing and analyzing the data, we found that among the nine sides diagnosed with IO through surgical exploration, four sides were diagnosed with EDO, and one side was diagnosed with EO-Cap through ultrasound. Of the nine sides initially diagnosed with IO by ultrasound, three were surgically diagnosed with EO-Cap, and two were confirmed as EDO. This highlights a notable finding: when evaluating for IO using ultrasound, it is frequently confused with EO-Cap and EDO. For EO-Cap, previous studies indicated [21, 22] that sperm produced in the testes enter the ductus epididymis within the epididymal caput but fail to migrate into the corpus, manifesting as net-like ectasia in the epididymal caput on ultrasound. The misdiagnosis likely resulted from EO-Cap presenting as net-like ectasia on ultrasound, while surgical exploration revealed sperm retention confined to the rete testis without epididymal migration. Additionally, three OA cases initially diagnosed as IO by ultrasonography were surgically confirmed as EO-Cap, with the absence of epididymal caput ectasia on imaging. It is possible that while sperm could enter the epididymal caput, the duration and degree of obstruction were insufficient to dilate the ductus epididymis within the epididymal caput. Given the diagnostic challenges, a key question is how to further improve the performance of ultrasound in diagnosing IO. Prior research has established that IO is a form of rete testis obstruction, where sperm accumulation increases intraluminal pressure, leading to dilation [21, 23]. Our study revealed significantly higher RTT in the IO compared to the EO, VDO, and EDO groups. This occurs as IO obstructs sperm transport from the seminiferous tubules to the epididymis, elevating rete testis pressure and subsequent dilation [9]. In contrast, EO, VDO, and EDO do not obstruct sperm flow within the testicles. Rocher et al [24] considered the rigidity of the rete testis through shear wave elastography, suggesting that substantial pressure is required to induce a measurable expansion of RTT. ROC analysis identified an optimal RTT cutoff of 3.0 mm for diagnosing IO. This cutoff yielded a high diagnostic performance with 88.9% sensitivity, 83.9% specificity, and an AUC of 0.842 (95% CI: 0.668–1.000). Therefore, while ultrasound images may show abnormal sonographic signs of obstruction, measuring RTT could further improve the diagnostic accuracy of IO.

Regarding EDO, our study showed that the diagnostic performance of transscrotal–transrectal ultrasonography in evaluating EDO was relatively high, but it was easily confused with IO. Previous studies [25] showed that when the diameter of the ejaculatory duct and the seminal vesicle was above 2.3 mm and 15 mm, respectively, especially when cysts appeared in the ejaculatory duct area, it indicated EDO. In this study, the misdiagnosis of IO as EDO on four sides occurred because transrectal ultrasonography revealed cysts in the ejaculatory duct area. Concurrently, transscrotal ultrasonography showed no signs of obstruction in the ipsilateral testis, epididymis, or scrotal vas deferens segment. However, on these four sides, the SVD was not greater than 15 mm, a previously suggested cutoff for EDO. The study found that while SVD significantly increased with EDO, its diagnostic performance was suboptimal, with a sensitivity of 62.5%, a specificity of 92.6%, and an AUC of 0.769 at a 12.5 mm cutoff. Despite the low sensitivity, the high specificity of the SVD measurement suggests it is useful for excluding EDO during the evaluation of IO, thereby improving the diagnostic accuracy for IO.

OA is most frequently caused by EO [14]. In our study, the proportions of EO and VDO were the highest, which is consistent with previous findings that OA predominantly involves the epididymis in Chinese populations, while it is more common in the vas deferens in Western populations [3, 26]. Our study showed that transscrotal–transrectal ultrasonography has a high diagnostic performance for locating OA obstruction in the epididymis and vas deferens. However, the patency and pregnancy rates after a VE procedure can vary depending on the specific location of the obstruction within the epididymis, whether it’s in the caput, corpus, or cauda [27, 28]. This highlights the importance of accurately determining the specific site of obstruction within the epididymis before performing VE. For this reason, we investigated whether ultrasound could be used to further pinpoint the exact location of the obstruction within the epididymis. The sensitivity of diagnosing EO-Cap, EO-Cor, and EO-Cau based on the appearance of net-like ectasia in the farthest part of the epididymis by ultrasound was found to be 40.0% (14/35), 65.2% (30/46), and 83.5% (91/109), respectively. This may be due to our determination of the specific obstruction site based on the appearance of obstruction at the farthest end of the epididymis on ultrasound. It is possible that sperm did not reach the farthest end of the epididymis, or, due to differences in the duration and degree of obstruction, the sperm went beyond the farthest end of the obstruction displayed on the ultrasound image. Therefore, the diagnostic accuracy in only using ultrasound images to determine the specific obstruction site of the epididymis was limited.

We attempted to further evaluate the diagnostic performance of measuring the thickness of the epididymal caput, corpus, and cauda, as well as the IDSSVD, in diagnosing the corresponding obstruction site. Our study found that ET-Cor, ET-Cau, and IDSSVD showed significant differences in the comparison of different obstructive sites. Therefore, we further evaluated the diagnostic performance of ET-Cor, ET-Cau, and IDSSVD in determining the corresponding obstructive site. We found that the overall diagnostic performance for EO-Cor at an ET-Cor cut-off of 4.1 mm and for EO-Cau at an ET-Cau cut-off of 7.5 mm was low. We believe the reasons are as follows: (1) EO-Cor causes ET-Cor thickening, and (2) the pressure of obstruction during EO-Cau and VDO continues to the epididymal corpus and cauda, causing dilation of the epididymal ducts in the epididymal corpus and cauda and, in turn, ET-Cor thickening. For the same reason, not only EO-Cau but also VDO caused ET-Cau thickening. The performance of ET-Cau was higher than that of ET-Cor in diagnosing EO-Cor may have been because EO-Cor, EO-Cau, and VDO could cause ET-Cor thickening, while only EO-Cau and VDO could cause ET-Cau thickening. Furthermore, our study found that when IDSSVD was 0.8 mm, the sensitivity, specificity, and AUC for diagnosing VDO were 81.0%, 100% and 0.949 (95% CI: 0.909–0.990), respectively, demonstrating excellent diagnostic performance. Du et al [2] found that IDSSVD above 1.5 mm was considered dilation, but our study was inconsistent with their study. In our study, when IDSSVD was 0.8 mm, dilation could be seen on ultrasound images, and it had high diagnostic performance for diagnosing VDO.

Our study has several limitations. First, its retrospective nature is a potential source of bias. Second, ultrasonography failed to visualize the pelvic segment of the vas deferens, a key part of the reproductive tract. Third, we lacked data on the duration of obstruction, which prevented us from assessing its impact on RTT imaging. To address these issues, future investigations should employ a prospective cohort design with serial ultrasonography. This approach will allow us to integrate obstruction duration as a core covariate in generalized estimating equation models to better understand its influence on imaging findings.

In conclusion, combined transscrotal–transrectal ultrasonography showed high diagnostic performance in determining the location of EO, VDO, and EDO in OA patients. RTT could further improve the diagnostic performance of evaluating IO. When it is difficult to identify the obstructive site of IO and EDO, the SVD could be useful for differential diagnosis between IO and EDO. Moreover, evaluating VDO through IDSSVD shows high diagnostic performance.

## Supplementary information

ELECTRONIC SUPPLEMENTARY MATERIAL

## Data Availability

We have provided a detailed description of the scope of application of the data in the materials and methods in the main text.

## Abbreviations

ANOVA: Analysis of variance
AUC: Area under the curve
CI: Confidence interval
EDO: Ejaculatory duct obstruction
EO: Epididymal obstruction
EO-Cap: Epididymal caput obstruction
EO-Cau: Epididymal cauda obstruction
EO-Cor: Epididymal corpus obstruction
ET-Cap: Epididymal caput thickness
ET-Cau: Epididymal cauda thickness
ET-Cor: Epididymal corpus thickness
FSH: Follicle-stimulating hormone
IDSSVD: Inner diameter of the scrotal segment of the vas deferens
IO: Intratesticular obstruction
IQR: Interquartile range
LH: Luteinizing hormone
OA: Obstructive azoospermia
ROC: Receiver operating characteristic
RTT: Rete testis thickness
SD: Standard deviation
T: Testosterone
TV: Testicular volume
VDO: Vas deferens obstruction
VE: Vasoepididymostomy
