# Supplementary material for: Localization of obstruction sites in obstructive azoospermia: role of combined transscrotal–transrectal ultrasonography
Source: Insights Imaging. 2025 Dec 2;16:269. doi: 10.1186/s13244-025-02143-x (PMC12672963; doi:10.1186/s13244-025-02143-x)
Supplement: Supplementary file 1 — ELECTRONIC SUPPLEMENTARY MATERIAL [file 13244_2025_2143_MOESM1_ESM.pdf]

Localization of obstruction sites in obstructive azoospermia: role of combined transscrotal-transrectal ultrasonography

ELECTRONIC SUPPLEMENTARY MATERIAL

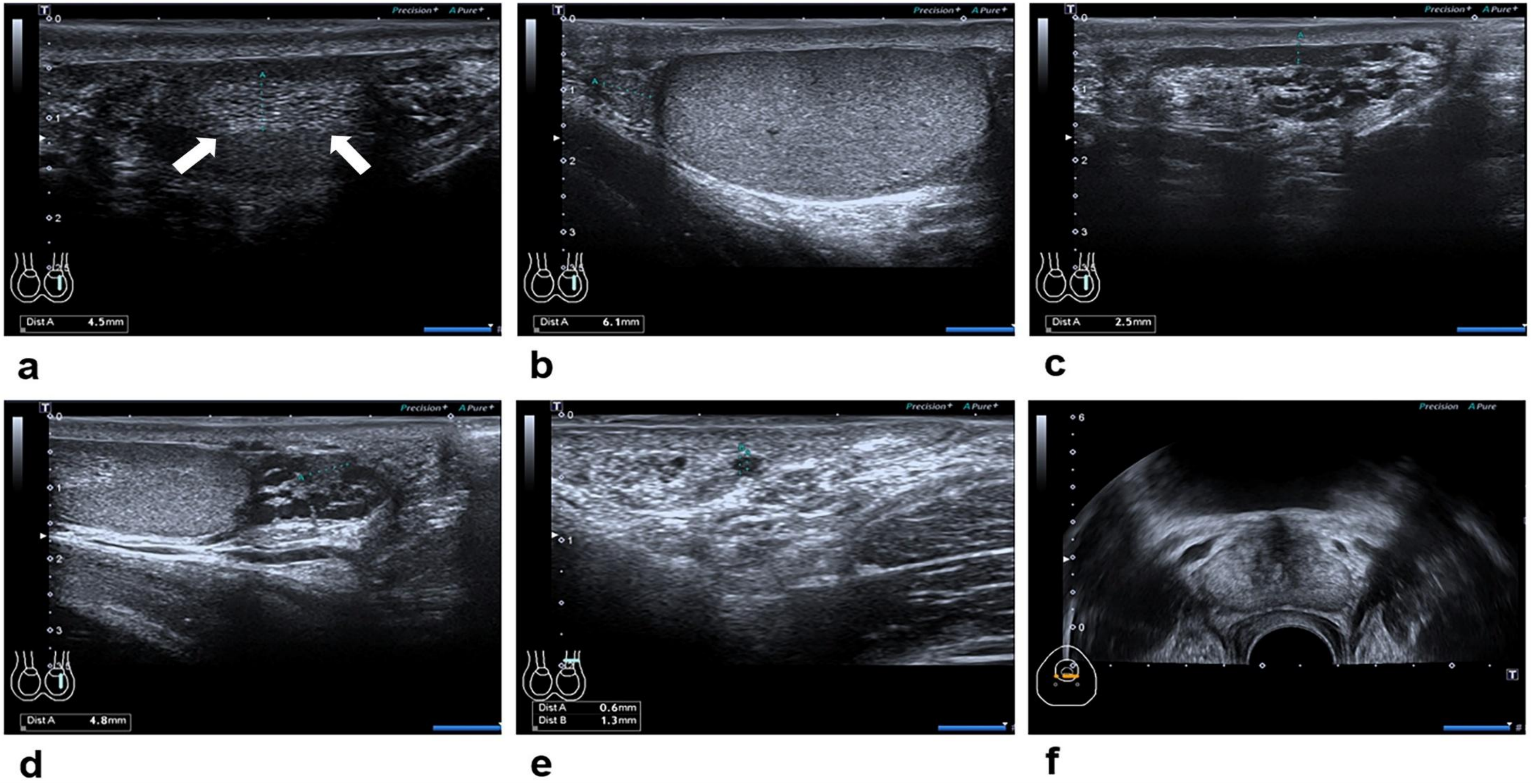

Supplement Figure 1. Intratesticular obstruction

An approximately 35-year-old male patient with obstructive azoospermia. The obstruction site was located within the left testicle. (a) The rete testis thickness of the left testicle was 4.5 mm, with net-like ectasia revealed by transscrotal ultrasonography (arrow). (b–d) The left caput, corpus, and cauda of the epididymis appeared normal on scrotal ultrasound without any ultrasound image features suggesting obstruction. (e) No dilation was caused by obstruction in the left scrotal segment of the vas deferens based on scrotal ultrasonography. (f) No obstructive factors were found in the ejaculatory duct area of the prostate, resulting in dilation of the ejaculatory duct area, as shown by transrectal ultrasonography.

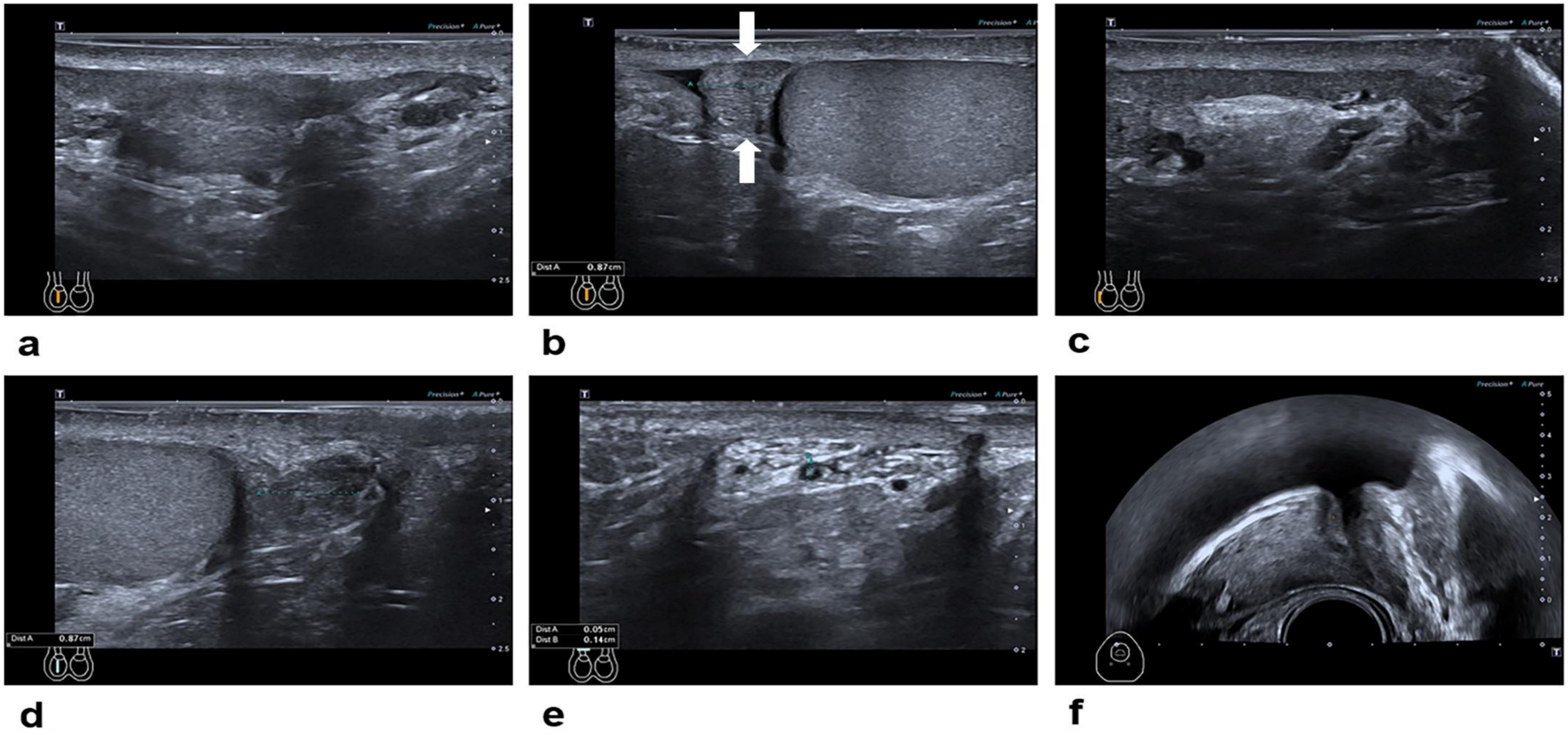

Supplement Figure 2. Epididymal caput obstruction

An approximately 25-year-old male patient with obstructive azoospermia. The obstruction site was located within the right epididymal caput. (a) The rete testis thickness was normal, without net-like ectasia on transscrotal ultrasonography. (b) The right epididymal caput showed net-like ectasia on transscrotal ultrasonography, and the epididymal caput thickness was 8.7 mm (arrow). (c-d) No dilation was caused by obstruction in the right epididymal corpus and cauda, as shown by transscrotal ultrasonography. (e) No dilation was caused by obstruction in the right scrotal segment of the vas deferens based on transscrotal ultrasonography. (f) No obstructive factors were found in the ejaculatory duct area of the prostate, resulting in the dilation of the ejaculatory duct area, as indicated on transrectal ultrasonography.

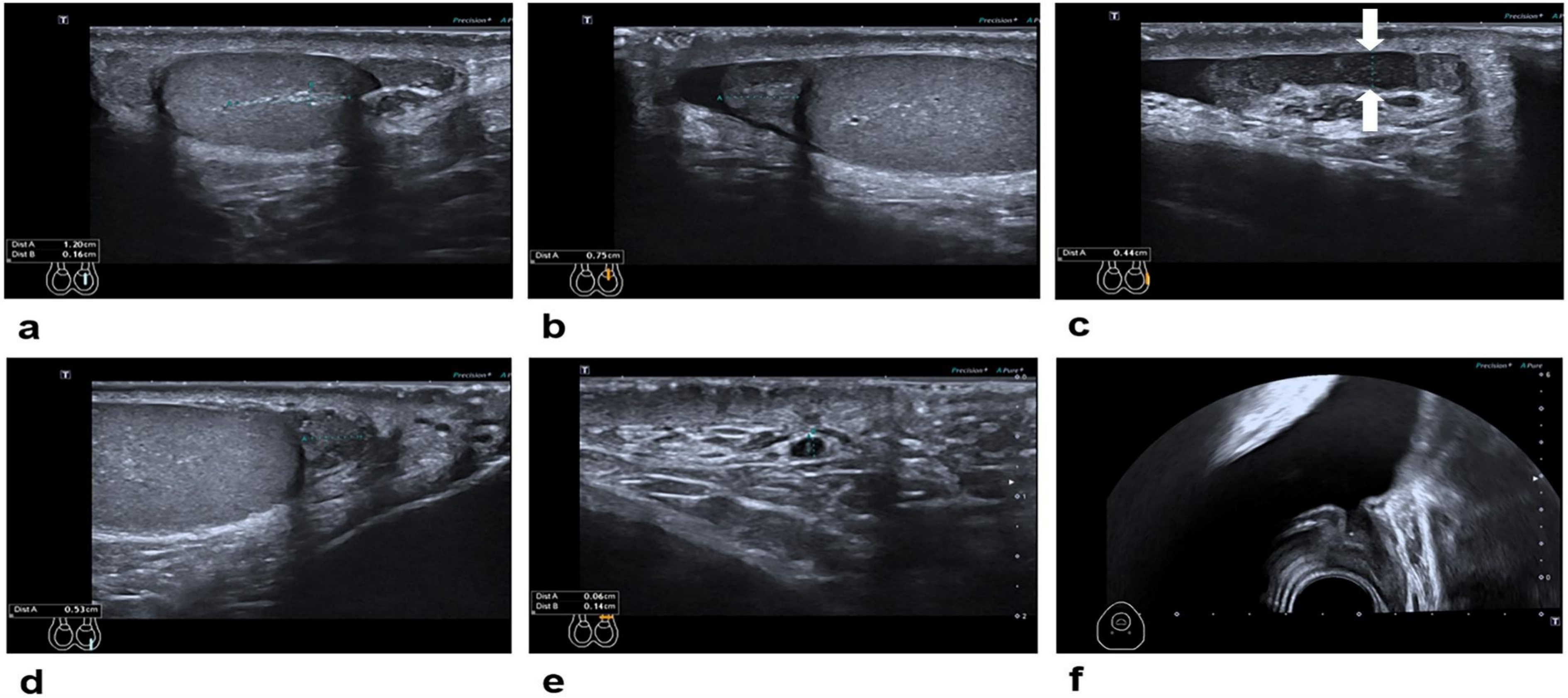

Supplement Figure 3. Epididymal corpus obstruction

An approximately 30-year-old male patient with obstructive azoospermia. The obstruction site was located within the left epididymal corpus. (a) The left rete testis thickness was 1.6 mm, without net-like ectasia on transscrotal ultrasonography. (b) The left epididymal caput appeared normal, without netlike ectasia on transscrotal ultrasonography. (c) The left epididymal corpus showed net-like ectasia on transscrotal ultrasonography (arrow). (d) The left epididymal cauda appeared normal, without net-like ectasia on transscrotal ultrasonography. (e) No dilation was caused by obstruction in the left scrotal segment of the vas deferens based on transscrotal ultrasonography, and the internal diameter of the scrotal section of the vas deferens was 0.6 mm. (f) No obstructive factors were found in the ejaculatory duct area of the prostate, resulting in the dilation of the ejaculatory duct area, as shown on transrectal ultrasonography.

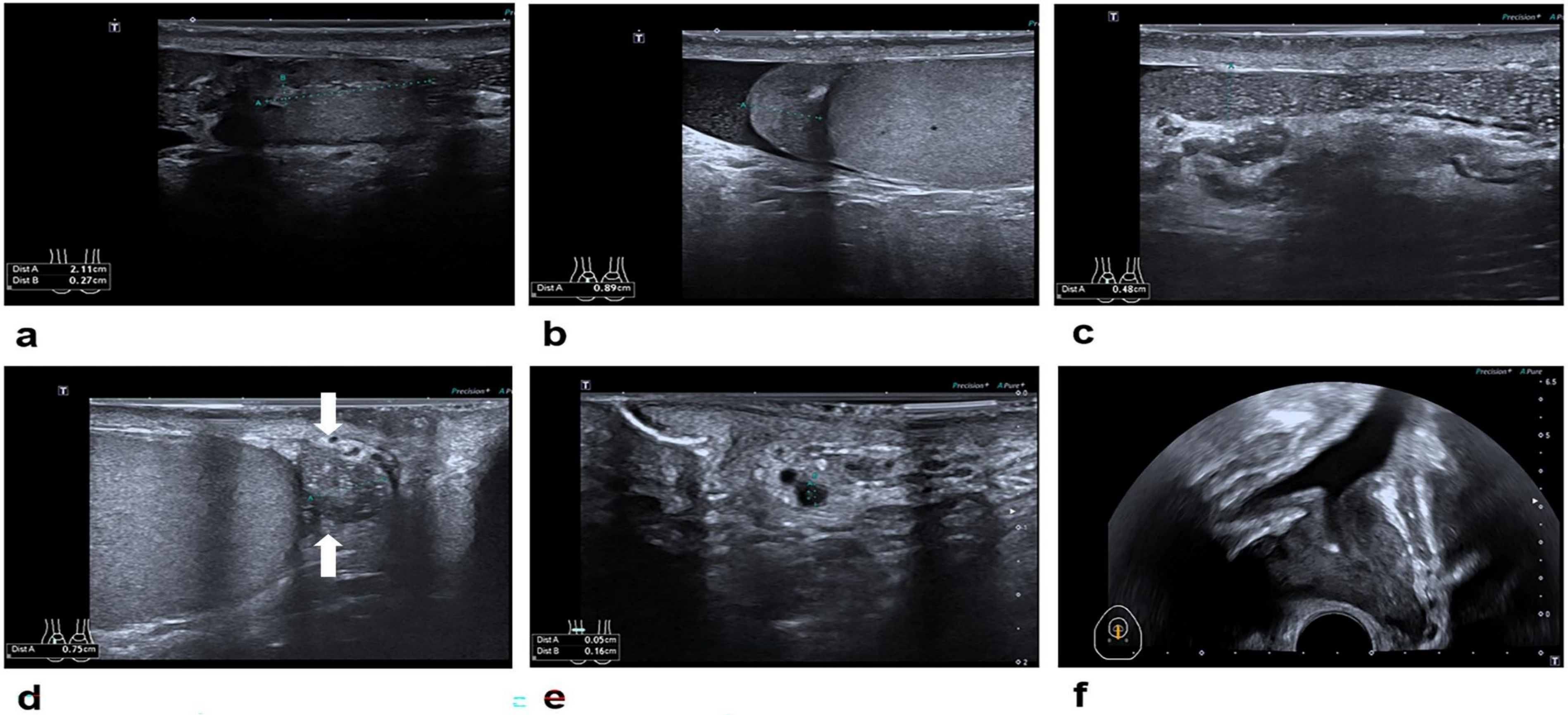

Supplement Figure 4. Epididymal cauda obstruction

An approximately 35-year-old male patient with obstructive azoospermia. The obstruction site was located within the right epididymal cauda. (a) The right rete testis thickness was 2.7 mm, without net-like ectasia on transscrotal ultrasonography. (b) The right epididymal caput showed net-like ectasia on transscrotal ultrasonography, and the right epididymal caput thickness was 8.9 mm. (c) The right epididymal corpus showed net-like ectasia on transscrotal ultrasonography, and the right epididymal corpus thickness was 4.8 mm. (d) The right epididymal cauda showed net-like ectasia on scrotal ultrasonography, and the right epididymal cauda thickness was 7.5 mm (arrow). (e) No dilation was caused by obstruction in the right scrotal segment of the vas deferens based on transscrotal ultrasonography, and the internal diameter of the right scrotal section of the vas deferens was 0.5 mm. (f) No obstructive factors were found in the ejaculatory duct area of the prostate, resulting in the dilation of the ejaculatory duct area, as shown on transrectal ultrasonography.

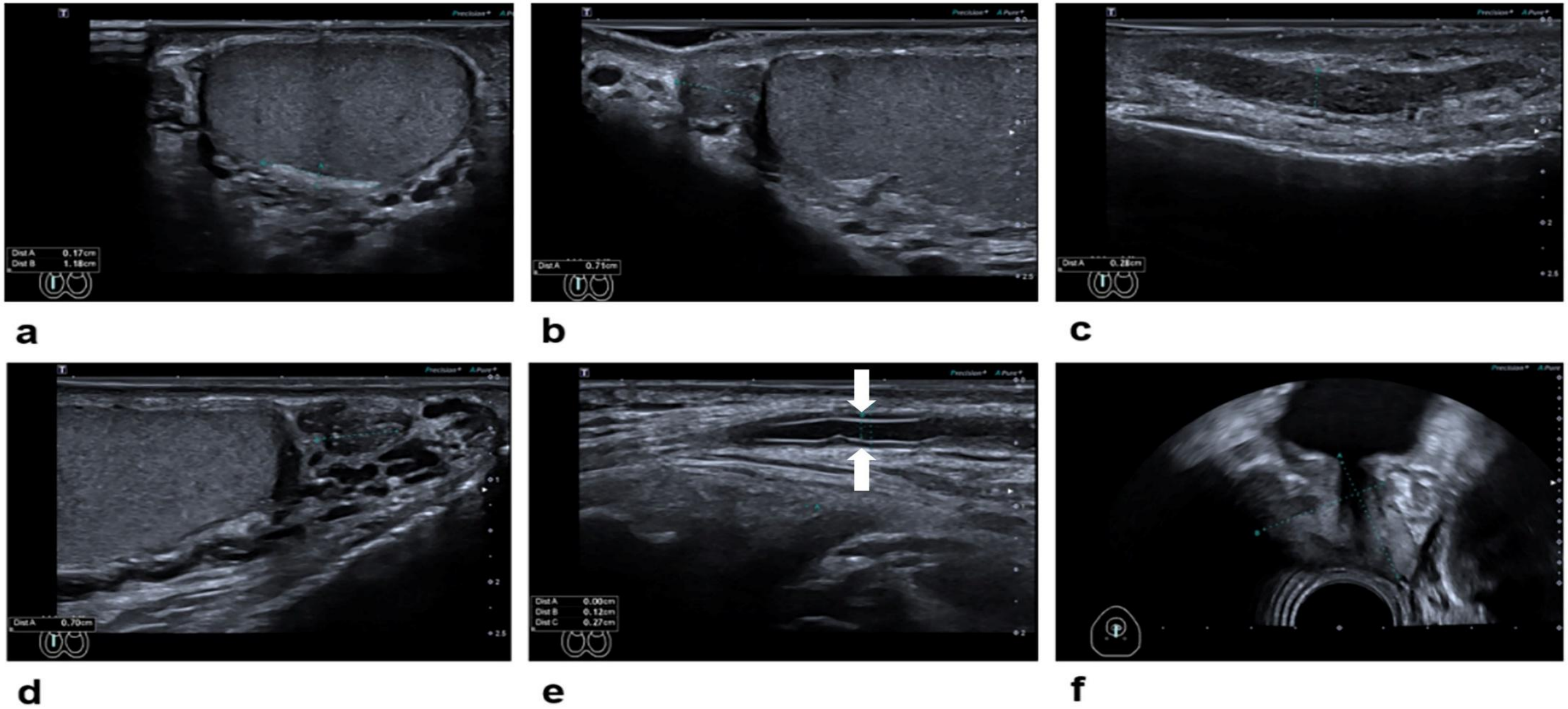

Supplement Figure 5. Vas deferens obstruction

An approximately 30-year-old male patient with obstructive azoospermia. The obstruction site was located within the right vas deferens. (a) The right rete testis thickness was 1.7 mm, without net-like ectasia on transscrotal ultrasonography. (b) The right epididymal caput appeared normal on transscrotal ultrasonography, and the right epididymal caput thickness was 7.1 mm. (c) The right epididymal corpus appeared normal on transscrotal ultrasonography, and the right epididymal corpus thickness was 2.8 mm. (d) The right epididymal cauda seemed normal on transscrotal ultrasonography, and the epididymal cauda thickness was 7.0 mm. (e) The right vas deferens scrotum segment was dilated on transscrotal ultrasonography, and the internal diameter of the right scrotal section of the vas deferens was 1.2 mm (arrow). (f) No obstructive factors were found in the ejaculatory duct area of the prostate, resulting in the dilation of the ejaculatory duct area, as shown on transrectal ultrasonography.

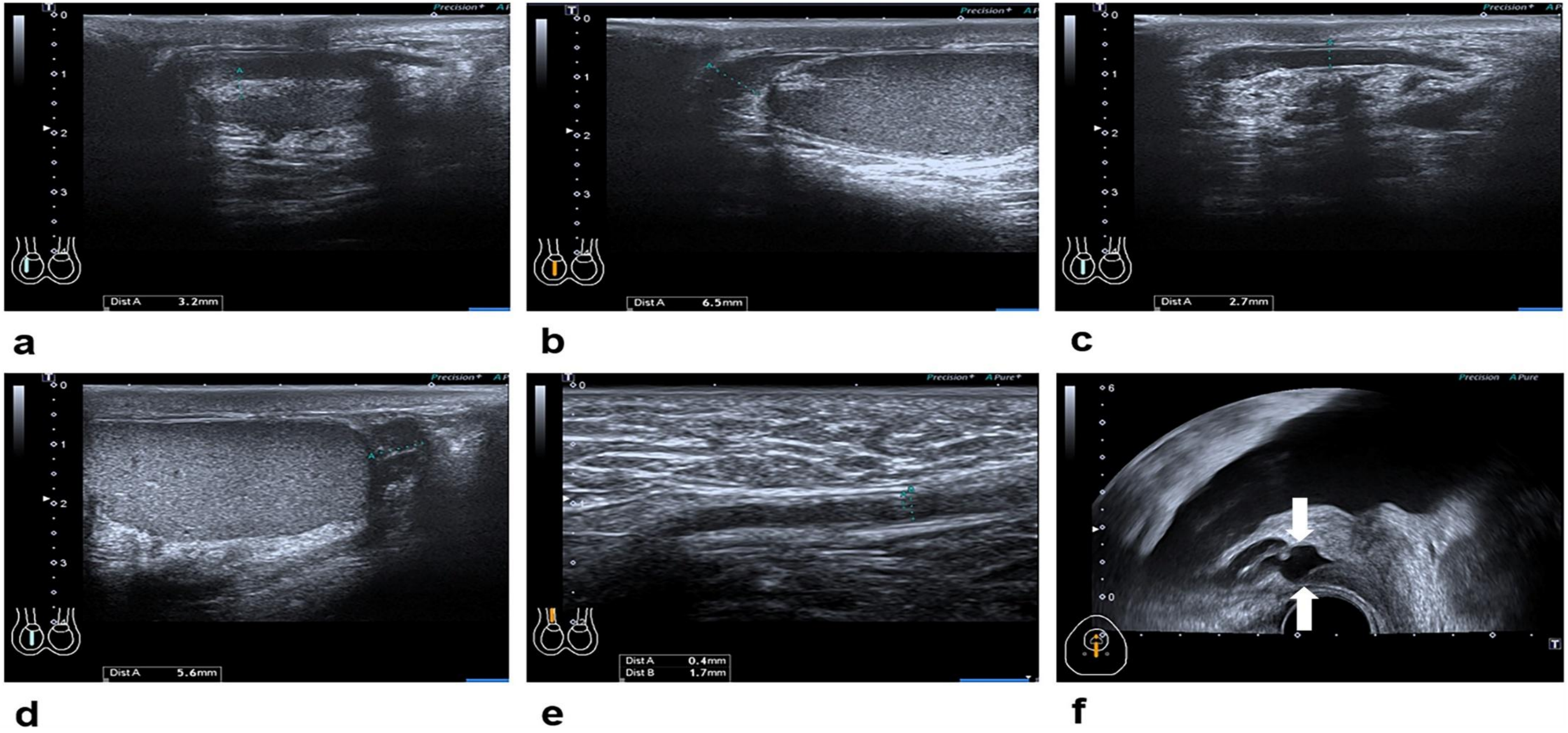

Supplement Figure 6. Ejaculatory duct obstruction

An approximately 40-year-old male patient with obstructive azoospermia. The obstruction site was located within the ejaculatory duct. (a) The right rete testis thickness was 3.2 mm, without net-like ectasia on transscrotal ultrasonography. (b-d) The right epididymal caput, corpus, and cauda appeared normal on transscrotal ultrasonography, without net-like ectasia on transscrotal ultrasonography. (e) No dilation was caused by obstruction in the scrotal section of the vas deferens based on transscrotal ultrasonography, and the internal diameter of the scrotal section of the vas deferens was 0.4 mm. (f) Cysts were seen in the ejaculatory duct area, leading to the obstruction of the area, as shown on transrectal ultrasonography (arrow).
